# A Potential Fungal Probiotic *Aureobasidium melanogenum* CK-CsC for the Western Honey Bee, *Apis mellifera*

**DOI:** 10.3390/jof7070508

**Published:** 2021-06-25

**Authors:** Chih-Kuan Hsu, Dun-Yan Wang, Ming-Cheng Wu

**Affiliations:** Department of Entomology, College of Agriculture and Natural Resources, National Chung Hsing University, Taichung 40227, Taiwan; orange970111@gmail.com (C.-K.H.); rocker840308@gmail.com (D.-Y.W.)

**Keywords:** bee bread, fungi, nutrient genes, antibacterial peptide genes

## Abstract

*Aureobasidium melanogenum* has been used as an animal feed additive for improving thehealth of pets, however, it has not yet been applied in honey bees. Here, a fungal strain CK-CsC isolated from bee bread pollen, was identified as *A. melanogenum*. Following characterizing CK-CsC fermentation broth, the 4-days fermentation broth (SYM medium or bee pollen) of the CK-CsC was used to feed newly emerged adult honey bees in cages under laboratory-controlled conditions for analysis of survival, gene expression of nutrient and antibacterial peptide, and gut microbiota of honey bees. It was found that the CK–CsC fermentation broth (SYM medium or bee pollen) is nontoxic to honey bees, and can regularly increase nutrient gene expression of honey bees. However, significant mortality of bees was observed after bees were fed on the supernatant liquid of the fermentation broth. Notably, this mortality can be lowered by the simultaneous consumption of bee pollen. The honey bees that were fed bee pollen exhibited more γ-Proteobacteria, Bacteriodetes, and Actinobacteria in their gut flora than did the honey bees fed only crude supernatant liquid extract. These findings indicate that *A. melanogenum* CK–CsC has high potential as a bee probiotic when it was fermented with bee pollen.

## 1. Introduction

Honey bee health and nutrition are closely related. Aside from honey, which mainly contains carbohydrates, honey bees feed on bee pollen, which provides comprehensive nutrition [1]. It has been found that owing to the improved environmental quality of land under the US Conservation Reserve Program (CRP) with regards to the abundance of diverse nectar plants, the nutritional and immune status of bee colonies on CRP land are better. Moreover, these bees have a high survival rate in the winter [2]. Feeding on high-quality pollen from diverse plants benefits bee physiology; for example, it supports the healthy development of the hypopharyngeal gland, the high expression of vitellogenin and immune genes, and even greater pathogen tolerance [3,4]. A favorable nutritional status protects bees from the numerous factors that threaten their health, both nonbiological (e.g., chemical drugs, electromagnetic waves, and climate change) and biological (e.g., pathogenic bacteria and *Varroa* mites) [5,6,7,8].

Honey bees make bee bread by mixing plant pollen with nectar and their saliva. Bee bread composition depends on the nectar sources, but in general, bee pollen contains approximately 13%–55%, 1%–20%, and 2%–60% carbohydrates, fat, and protein, respectively [9,10,11,12,13]. After its transport back to the hive, pollen is stored in the honeycomb and made into bee bread. Bee bread contains an abundance of microorganisms, including bacteria and fungi. Bacterial communities in bee bread, which typically include *Lactobacillus* spp. and *Acinetobacter* spp., secrete organic acids that help preserve the bee bread [14,15]. As for fungi, *Aspergillus* spp., *Cladosporium* spp., *Penicillium* spp., *Rhizopus* spp., and *Aureobasidium* spp. have been observed in bee bread. These fungi can secrete multiple types of extracellular enzymes to convert carbohydrates, fats, and proteins into organic acids, antibiotics, and other metabolites [16]. Fungi that are typically present in bee bread assist in its preservation and the conversion of its nutrients [15,17,18].

The use of probiotics for honey bees to improve bee health has recently gained popularity [19,20,21,22]. In honey bees, probiotics have been found that not only improve the gut metabolism (e.g., carbohydrate breakdown), resulting in increased body weight [23,24,25], but also reduce diseases by preventing intestinal infection and stimulating host immune responses [26,27]. Most of probiotics used/ tested in honey bees belong to lactic acid bacteria which show beneficial effects on honey bees [22]. Only few of probiotics tested in honey bees possess fungal strains. For example, a yeast strain was included in a commercial probiotic mixture can increase colonies’ strength and help honey bees against the *Nosema* spp. [28]. The fungal strains, *Botrytis cinerea*, *Cladosporium* sp. and *Colletotrichum acutatum* are assumed to have a nutrition value for honey bees [29].

In Taiwan, pollen from tea tree (*Camellia sinensis*) has the highest production output of approximately 150 tons per year [30]. We are interested in examining the microbial flora within the bee bread made from this type of pollen. This study centered on fungi. During the screening process, *Aureobasidium melanogenum* was observed. The fungus, which is common in the natural world, has been isolated from soil, mud, rotted wood, limestone, and water as well as from wet environments such as bathroom surfaces. It has been found in honey [31,32,33,34,35]. Studies have demonstrated that exopolysaccharides (EPSs) produced by *Aureobasidium melanogenum* promote anti-inflammatory and anti-tumor activities in mammals [36,37] as well as cholesterol metabolism [38]. This explains the use of this fungus in animal feed additives [39]. Furthermore, this fungus secretes numerous enzymes, including amylase, cellulase, proteinase, lipase, xylanase, mannanase, polygalacturonase, and transferases [32,33,34,40], which are used widely in the food processing industry [41]. Despite the development potential of *A. melanogenum* in animal feed, research on its biological effects on honey bees is lacking. Therefore, we conducted a preliminary discussion of the fermentation characteristics of the CK–CsC and further assessed the effects of the CK-CsC fermentation broth on honey bees via monitoring the expression of genes encoding nutrient related genes, i.e., major royal jelly protein 1 (*mrjp1*) and vitellogenin (*vg*), and immune related genes, i.e., antibacterial peptide genes apidaecin and hymenoptaecin. To the best of our knowledge, this is the first study to explore the effects of *A. melanogenum* CK–CsC, isolated from bee bread, on honey bee.

## 2. Materials and Methods

### 2.1. Honey Bees

The honey bee (*Apis mellifera*) colonies were maintained in the apiary at National Chung Hsing University. Each bee colony contained a young egg-laying queen, had a working population of eight frames of comb with eggs, larvae, pupae, and food (i.e., honey and bee bread). The healthy honey bee colonies were maintained in accordance with standard beekeeping guidelines. For evaluation of fungi fermentation broth effect on honey bees, the sealed brood frames were removed from the bee colony and maintained in the insect growth chamber (temperature: 33 ± 1 °C, relative humidity: 60% ± 10%, dark) until the pupae emerged as adult bees. The emerging worker bees were then subjected to further experiments.

### 2.2. Isolation and Preservation of Fungal Strains in Camellia sinensis Bee Bread

Bee bread produced from *C. sinensis* pollen was obtained from the honeycombs, and 200 mg portions were homogenized in 1 mL sterile water. Next, an inoculation loop was used to sample the pollen solution and create a streak culture in potato dextrose agar (PDA) containing 35 mg/L of chloramphenicol. The streak culture was incubated at 28 °C for 48 h; this was followed by a second round of streaking. The new growth colonies were then examined and confirmed to be of a single type—that is, a pure strain. After the strain had sporulated, the surface of the colony was washed with phosphate buffer (pH 7.0), and impurities and hyphae were removed from the collected spore mixture using a sterile, defatted cotton swab. The obtained spore filtrate, which contained 20% glycerol, was divided into separate samples and stored at −80 °C.

### 2.3. Molecular Identification of FUNGAL Strains

The fungal DNA was extracted using PureLink DNA Purification Kit (Thermo Fisher Scientific, Rockford, IL, USA) according to the manufacturer’s instructions. Specific primers ITS1 and ITS4 (Supplementary Table S1) were used to perform the polymerase chain reaction (PCR) and amplify the gene fragments in the internal transcribed spacer region (ITS; including 5.8S rDNA) [42]. The amplification settings were as follows: Stage 1 involved one cycle of denaturing at 94 °C for 5 min. Stage 2 involved 35 cycles of denaturing at 94 °C for 30 s, 50 °C for 20 s, and 72 °C for 45 s. Each PCR sample had a total volume of 25 μL, contained 10× PCR buffer, 0.1 μM of primer pair, 10 mM dNTP, 25 ng of DNA, and 0.25 μL of Taq DNA polymerase (Takara Bio Inc., Shiga, Japan). The amplified ITS gene fragments were then cloned into a pGEM-T vector (Promega, Madison, WI, USA) for bidirectional sequencing. The obtained sequences were analyzed using Vector NTI software V10 (Invitrogen), and the nucleic acid sequences were deposited into the GenBank database established by the US National Center for Biotechnology Information (NCBI). The phylogenetic tree was constructed by using MEGA X [43] with a neighbor-joining method [44]. Bootstrapping was performed for 1000 replicates, and the evolutionary distances were computed using the Kimura two-parameter model [45,46].

### 2.4. Analysis of the CK–CsC Fermentation Broth

A total of 10^7^ spores of the CK–CsC strain was inoculated into 10 mL of YPD medium (a seed culture, 10 g of yeast extract, 20 g of peptone, 100 g of glucose, and 0.5 g of chloramphenicol in 1 L) [33] and incubated in a shaking incubator at 28 °C for 1 day. Then 10 mL of seed culture was added to 90 mL of SYM culture medium (140 g of sucrose, 3 g of yeast extract, 0.6 g of (NH_4_)_2_SO_4_, 5 g of K_2_HPO_4_, 1 g of NaCl, and 0.2 g of MgSO_4_·7H_2_O in 1 L) [33], which was incubated with shaking at 28 °C and sampled after incubation for 24, 48, 72, 96, and 120 h, respectively. The pH, protein content, biomass, and EPS output of the samples were then determined. The pH values were measured by applying a pH meter (Sartorius PB-10, Sartorius Lab Instruments GmbH & Co. KG, Goettingen, Germany) to the supernatant liquid obtained from the centrifugation of 10 mL of the fermentation broth. Protein content was measured by applying the Pierce BCA Protein Assay Kit (Thermo Fisher Scientific, Waltham, MA, USA) to 1 mL of the supernatant liquid. To measure the biomass, 10 mL of fermentation broth was freeze dried, and the weight of the control group (i.e., 10 mL of powder from freeze-dried SYM culture not containing spores) was subtracted from the weight of the obtained powder. The EPS output was the dry weight of the freeze-dried precipitate from the mixture of the 10 mL of supernatant liquid with 20 mL of cool absolute ethanol [33]. The characteristic analysis of the fermentation broth was performed in three replicates.

### 2.5. Effects of the CK–CsC SYM Fermentation Broth on Honey Bee

#### 2.5.1. Effects of the CK–CsC SYM Fermentation Broth on the Honey Bee Survival Rate

The experiment was performed on three groups of 50 newly emerged adult honey bees, which were given 50% sucrose syrup (S), SYM broth that had fermented for 4 days (F), and supernatant liquid from that fermentation broth (FS), respectively. The honey bees were placed in a transparent acrylic cage (15 cm × 10 cm × 15 cm) that were transferred to a growth chamber with no light in which the temperature and relative humidity were maintained at 33 ± 1 °C and 60% ± 10% [47]. The syrup or fermentation broth was replaced every 3 days, and the bees were observed for 1 week, during which the mortality rate was recorded and the dead bees were removed. This experiment was conducted three times using three honey bee colonies.

#### 2.5.2. Effects of the CK–CsC SYM Fermentation Broth on Gene Expression of Bees

Due to the supernatant liquid of the SYM fermentation, broth is toxic to adult honey bees, only the SYM fermentation broth was assessed with 50 newly emerged adult honey bees for its effects on gene expression. The SYM medium or fermentation broth given to the honey bees was replaced every 3 days. On the seventh day, the bees were anesthetized with CO_2_, and 10 honey bees were randomly selected for dissection, specifically to purify and extract RNA from the head and abdomen. This experiment was conducted three times using three honey bee colonies.

### 2.6. Effects of Polyfloral Bee Pollen Fermented by CK–CsC on Honey Bee

The polyfloral bee pollens, used in this study, were collected with a pollen trap in the university apiary from March to May (spring season in Taiwan) [47]. A total of 10^7^ CK–CsC spores were inoculated into YPD medium that was then incubated with shaking for 1 day at 28 °C. Afterward, 10 mL of seed culture was mixed with 30 g of bee pollen, and fermented at 28 °C for 4 days.

#### 2.6.1. Effects of the Fermented Pollen on the Honey Bee Survival Rate

Three groups of 50 newly emerged adult honey bees were given 50% sucrose syrup and polyfloral pollen (S + P), 50% sucrose syrup and pollen mixed with SYM medium (S + SYM-P), and 50% sucrose and CK-CsC fermented pollen (S + CK–CsC-P), respectively. The syrup and pollen cakes were replaced every 3 days, and the bees were observed for a week, during which the mortality rate was recorded and the dead bees were removed. This experiment was conducted four times using four honey bee colonies.

#### 2.6.2. Effects of the Fermented Pollen on Gene Expression of Bees

After 6 days of feeding, 10 bees from the S + SYM-P and S + CK–CsC-P groups were randomly selected for dissection, specifically to purify and extract RNA from the head and abdomen. This experiment was conducted four times using four honey bee colonies.

### 2.7. Purification and Extraction of Total RNA of Honey Bees

The purification and extraction of honey bee RNA were performed using TRIzol reagent and the PureLink RNA Mini Kit (Thermo Fisher Scientific, Waltham, MA, USA) according to the manufacturer’s instructions. After the RNA was extracted, the residual DNA in the samples was removed using a TURBO DNA-free Kit (Thermo Fisher Scientific). The purified RNA samples were then quantified using a Qubit fluorometer (Thermo Fisher Scientific) and stored at −80 °C.

### 2.8. Purification and Extraction of Gut DNA of Honey Bees

DNA was extracted from the midguts and hindguts of 10 honey bees using the PureLink DNA Purification Kit (Thermo Fisher Scientific, Waltham, MA, USA). After purification, the DNA was quantified using a Qubit fluorometer (Thermo Fisher Scientific) and stored at −20 °C.

### 2.9. Gene Expression Measurement Using Reverse Transcription-Quantitative PCR (RT-qPCR)

The iScript cDNA Synthesis Kit (Bio-Rad, Hercules, CA, USA) was used to reverse transcribe 1 μg of total RNA into cDNA. Next, qPCR was performed to determine the expression of nutrient genes *mrjp1* and *vg* and antibacterial peptide genes apidaecin and hymenoptaecin. The primers used are presented in Supplementary Table S2 [23,47,48,49]. Each qPCR assay was conducted in a 96-well plate, and every 20 μL of reaction solution contained 10 μL of 2 × iQ SYBR Green Supermix (Bio-Rad, Taipei, Taiwan), 2.5 μL of 1.6 μM of each gene-specific primer, and 5 μL of diluted cDNA. After the reaction agents were added, the 96-well plate was placed inside a CFX Connect Real-Time System (Bio-Rad) and allowed to react. The reaction conditions were one cycle at 95 °C for 3 min, followed by 40 cycles at 95 °C for 10 s and at 59 °C for 30 s. For each cycle, the fluorescent reaction signals were detected, and Bio-Rad CFX Maestro software was used to collect and normalized to the reference gene rpS18 [50]. The relative gene expression data were analyzed using the 2^−ΔΔ*C*T^ method [51]. Each RT-qPCR assay involved at least three independent biological replicates with three technical repetitions.

### 2.10. Gut Microflora Quantification with qPCR

The primers used are presented in Supplementary Table S1 [52,53]. The primer pairs were used to determine the abundance of total bacteria, *Lactobacillus kunkeei,* and the amounts of bacteria from five phyla, namely α–Proteobacteria, γ–Proteobacteria, Bacteriodetes, Firmicutes, and Actinobacteria in the bee guts. Every 20 μL of qPCR reaction solution contained 10 μL of 2 × iQ SYBR Green Supermix, 2.5 μL of 1.6 μM of each gene-specific primer, and 5 μL of diluted gut DNA. The cyclic procedure included one cycle at 95 °C for 3 min, followed by 40 cycles at 95 °C for 10 s and at 55–59 °C for 30 s. For each cycle, the fluorescent reaction signals were detected, and Bio-Rad CFX Maestro software was used to collect and normalized to the reference gene actin [50]. The relative bacterial abundance was analyzed using the 2^−ΔΔ*C*T^ method [51]. Each qPCR assay involved at least four independent biological replicates (10 guts for each repeat) and three technical repetitions.

### 2.11. Statistical Analysis

Analyses were performed using SAS software, Version 9.4 of the SAS System (SAS Institute Inc., Cary, NC, USA) and graphs were created using SigmaPlot 14.0 software (Systat Software Inc., San Jose, CA, USA). Honey bee survival rate was analyzed using a Kaplan-Meier survival curve, and the significant differences among groups were subjected to Log-Rank test and Holm-Sidak test for multiple comparisons when applicable. The data on gene expression and gut microflora changes were analyzed using Mann–Whitney U tests.

## 3. Results and Discussion

### 3.1. Isolation and Molecular Identification of Fungi from Pollen Bee Breads

**Figure 1:**
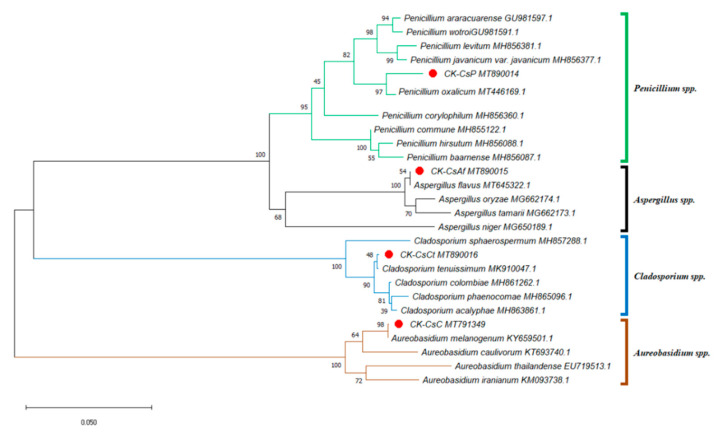
Phylogenetic tree of the isolated fungi constructed using the sequences of internal transcribed spacer regions (incl. 5.8S rDNA) (ITS). The isolated fungi were labeled as a red spot. Sequences for the ITS phylogenetic analysis were obtained from the GenBank database for the following strains: *Aspergillus flavus, Aspergillus niger, Aspergillus tamarii, Aspergillus oryzae, Aureobasidium caulivorum, Aureobasidium iranianum, Aureobasidium thailandense, Aureobasidium melanogenum, Cladosporium acalyphae, Cladosporium colombiae, Cladosporium phaenocomae, Cladosporium* sphaerospermum, *Cladosporium tenuissimum, Penicillium araracuarense, Penicillium baarnense, Penicillium corylophilum, Penicillium commune, Penicillium hirsutum, Penicillium vavanicum var. javanicum, Penicillium levitum, Penicillium oxalicum, Penicillium wotroi.* The GenBank accession number is included after the fungal scientific name. The tree was constructed using the neighbor-joining method and tested by bootstrapping with 1000 replicates of data. Percentages are reported at nodes, and the scale bar represents 0.05% sequence divergence.

Taiwan’s location in subtropical and tropical climate zones explains its abundant flora, consisting of over 500 nectar plants for honey bees [54]. With tea being a widely planted crop, pollen from *C. sinensis*, which is commonly collected by local apiarists, accounts for more than one third of Taiwan’s bee pollen output [30]. This fueled our interest in understanding the fungal microbial composition of bee bread made from the storage of this type of bee pollen in honeycombs. We isolated at least ten fungal strains from the bee bread, but only four fungal strains could be identified according to the results from the ITS sequence analysis (including 5.8S rDNA) of approximately 600 base pairs with genetic sequence similarities of 98% or higher. Furthermore, the ITS phylogenetic tree was drawn by analyzing 26 sequences. Aside from CK–CsAf, CK–CsC, CK–CsCt, and CK–CsP, four *Aspergillus* spp. sequences, four *Aureobasidium* spp. sequences, five *Cladosporium* spp. sequences, and nine *Penicillium* spp. sequences were obtained from the NCBI dataset. The results indicate that strain CK–CsAf (MT890015) was a monophyletic group among *Aspergillus* spp., CK–CsC (MT791349) was a monophyletic group among *Aureobasidium* spp., CK–CsCt (MT890016) was a monophyletic group among *Cladosporium* spp., and CK-CsP (MT890014) was a monophyletic group among *Penicillium* spp. (Figure 1). Furthermore, the CK–CsAf and CK–CsC sequences were a 100% match with *Aspergillus flavus* and *A. melanogenum*, respectively. The CK–CsCt and CK–CsP sequences were a 99.8% and 98.7% match with *Cladosporium tenuissimum* and *Penicillium oxalicum*, respectively (Supplementary Table S3). These four strains have all been recorded to be isolated from hive-stored bee bread, and these results are consistent with previous studies on fungi in bee bread made by *A. mellifera* [15,16].

*A. flavus, C. tenuissimum,* and *P. oxalicum* can often be isolated from the air [55] and are opportunistic animal pathogens [56,57,58,59]. Previous studies have found that *Aspergillus* spp. and *Penicillium* spp. can repress the growth of honey bee pathogenic fungi [60]. However, honey bees’ consumption of *A. flavus* conidia was found to express a reduced survival rate [59]. On the other side, Parish et al. observed that the addition of *Cladosporium* sp. spores to pollen significantly increased bees’ survival rates and extended their life cycles. The researchers postulated that the cause may have been linked to the existence of β-glucan on the spore walls [29]. *A. melanogenum*, which is widespread in nature, is a fungus that can produce EPSs and secrete various extracellular enzymes. It is widely used in the food processing industry and is a component of animal feed [31,34,39,41]. Since *A. flavus, C. tenuissimum,* and *P. oxalicum* have been studied in honey bees, *A. melanogenum* would be a good animal probiotic but has not been investigated in honey bees. Therefore, we focus on exploration of the effects of *A. melanogenum* CK–CsC on survival and gene expression of honey bees.

### 3.2. Characterization of the Aureobasidium melangenum CK-CsC Fermentation Broth

By day 5 of the preliminary experiments involving the fermentation of CK–CsC in a PDA medium, the EPSs produced were at a relatively low concentration of 4.1 mg/mL. The culture medium was later changed to a SYM medium, which has a higher sucrose concentration (140 g/L); as a result, the EPS output by day 5 was also higher, at 15.8 mg/mL (Table 1). However, this output was lower than the typical output of *A. melangenum* for industrial use (30–80 mg/mL) [61]; this may be related to the expression of polysaccharide synthesis genes of the fungal strain [33]. Regarding fermentation and cultivation in the SYM medium, the pH value of the fermentation broth gradually decreased over time, from 7.5 on day 1 to 3.1 by day 5. This trend is comparable to that observed in multiple fungal strains isolated from bee bread pollen, which create an acidic environment that inhibits microbial growth and preserves the bee bread [14,15]. The biomass increased from 21.4 mg/mL on day 1 to 43.4 mg/mL by day 5. As for the protein content, it nearly quadrupled from 5.5 to 21.6 mg/mL over the same period (Table 1). Furthermore, the activity of pectinase and cellulase in the CK–CsC strain can be observed in the pectinase and cellulase screening agar (Supplementary Figure S1) and might play a role in degrading the pectin and cellulose on pollen wall surfaces.

### 3.3. Effects of the CK–CsC SYM Fermentation Broth on Honey Bee

The CK–CsC strain was fermented in a sucrose-rich SYM culture medium to facilitate the production of EPSs [33]. The effects of CK–CsC on honey bees were assessed through the examination of this fermentation broth and the crude EPS extract.

**Figure 2:**
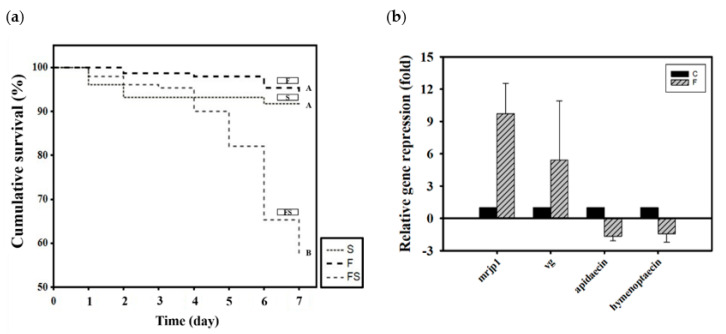
The effect of CK-CsC SYM fermentation broth on honey bees. (**a**) The cumulative survival of honey bees after 6-day feeding treatments. New emerged bees in groups of 50 bees fed with 50% sucrose syrup (S), CK-CsC SYM fermentation broth (F) and supernatant of CK-CsC SYM fermentation broth (FS). Each point represents the cumulative survival rate (N = 4 cages from four colonies). Different letters are significantly different (*p* < 0.05) accordingly to the Holm-Sidak test. (**b**) Gene expression profile of honey bees after 6-day feeding treatments. C: control, bees feeding with SYM broth; F: bees feeding with CK-CsC SYM fermentation broth. The relative gene expression was analyzed using the 2^−ΔΔ*C*T^ method. Data represent the mean of three repeats, with error bars indicating the standard deviation.

Newly emerged adult bees were divided into three groups according to how they were fed CK–CsC fermented in a SYM culture medium: 50% sucrose syrup (S), SYM fermentation broth (F), and the supernatant liquid of the SYM fermentation broth (FS). After 6 days, the survival rates in the S, F, and FS groups were 94%, 94%, and 57%, respectively (χ^2^_2, 450_ = 74.44, *p* < 0.05; Figure 2a). The SYM fermentation broth (F) was not toxic to the honey bees, and their survival rate was the same as that of the group that was fed syrup (S). However, the supernatant liquid of the SYM fermentation broth was toxic to the honey bees; after 5 days of feeding, the mortality rate rose to 20%. By day 7, the mortality rate had exceeded 40%. Honey bee mortality was initially presumed to be ascribable to the EPS in the supernatant liquid under the assumption that its molecular weight may have been excessively high, resulting in indigestion. Barker found that some sugars, including arabinose, xylose, galactose, mannose, lactose, melibiose, and raffinose are toxic to honey bees, shortening their lifespan if ingested [62]. However, no studies have attributed EPS to honey bee mortality. The reason for the high survival rate among the F group honey bees speculated is that the presence of CK–CsC mycelium in fermentation broth can secrete various extracellular enzymes to metabolize and utilize high-molecular-weight EPSs in culture media [16].

To further investigate the lethality of EPSs to honey bees, we used alcohol to extract the EPSs from the supernatant of the CK–CsC fermentation broth [33], feeding the crude extract to the honey bees at a 1% concentration. After 3 days, the mortality was 40%; by day 7, the mortality rate was over 80% (χ^2^_3, 800_ = 419.38, *p* < 0.05; Supplementary Figure S2). Therefore, we concluded that EPS extract was mainly responsible for the honey bee mortality. Notably, this mortality rate was lowered by the honey bees simultaneously feeding on bee bread. The survival rate of the honey bees feeding on bee bread was 90% on day 3 and 70% on day 7 (the E + P group in Supplementary Figure S2). Although we did not examine the lethal mechanism of the crude EPS extract, we assume that the presence of CK–CsC mycelium and microflora obtained from bee bread helped the honey bees break down the extract. Although studies have not indicated that honey bees can die from feeding on polysaccharides, they have noted that polysaccharides derived from honey bee-collected pollen, such as cellulose and pectin, must be broken down by honey bee gut microbes for effective utilization [63,64,65,66].

After the nontoxicity of the CK–CsC fermentation broth in the SYM medium was confirmed, its effects on gene expression were assessed. Relative to the honey bees that were only given SYM culture medium, those that fed for 6 days on the broth that was fermented for 4 days expressed 10 and 5 times more *mrjp1* (Z = −1.7457, *p* > 0.05) and *vg* (Z = −1.7457, *p* > 0.05; Figure 2b), respectively. Statistically, the differences of both gene expressions were not significant between SYM medium and fermented broth treatments, this may be due to the wide variation responses of bees from the three tested colonies. Nevertheless, all bees from the three colonies responded positively to CK–CsC fermentation broth which upregulates the *mrjp1* expression of bees by 2.5, 5.9 and 20.8 fold in three tested colonies, respectively, and the *vg* expression of bees by 9.2, 1.9 and 5.0 fold in three tested colonies, respectively. This stimulation effect was inferred to be caused by the progressive growth of CK–CsC over the fermentation period and the gradual increase in the protein content of the fermentation broth (Table 1). In honey bees, increased protein intake stimulates the expression of nutrient genes. Such results from fungal fermentation broth are similar to the previous reports of the positive effects of fermentation broth containing lactic acid bacterial strains on honey bee nutrition [23,48]. However, the expression of the immune genes apidaecin and hymenoptaecin were not significantly increased by fermentation broth consumption; in fact, it was suppressed 1.5 times (Z = 0.4364, *p* > 0.05 for apidaecin; Z = 0.8729, *p* > 0.05 for hymenoptaecin; Figure 2b). This suppression contrasts with past findings that probiotics can stimulate the expression of antibacterial peptides in honey bees [26,27,48]. The exact effects of CK-CsC fermentation broth on bees’ immune system needs to be further addressed in future.

### 3.4. Effects of the CK–CsC Fermented Pollen on Honey Bee

Pollen is known to be a vital food for honey bees, improving their nutritional status and bolstering their immune function [67,68,69]. The present strain of CK–CsC, isolated from bee bread, can secrete extracellular enzymes such as pectinase and cellulase and is speculated to be able to degrade polysaccharides on pollen cell walls. In using the CK–CsC strain for pollen fermentation, the pollen changed from a solid to a liquid with increasing viscosity (Supplementary Figure S3). The fungal strain was surmised to degrade the wall structure and thereby change the nutrient composition. This is consistent with findings from studies on fungal contributions to nutrient conversion in bee bread [16,18]. Therefore, pollen was used as the base of the broth, which was fermented over 4 days, to assess the effects of CK–CsC on the honey bees.

**Figure 3:**
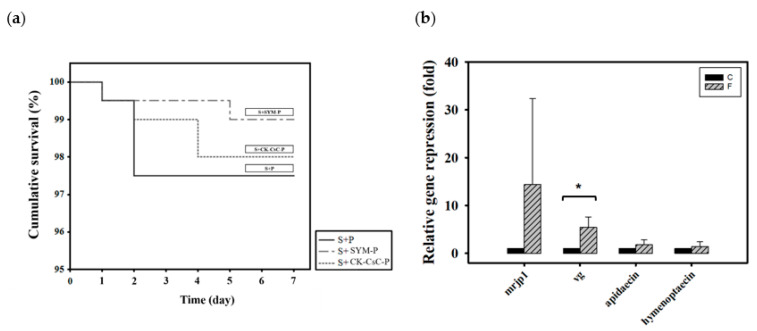
The effect of CK-CsC fermented pollen on honey bees. (**a**) The cumulative survival of honey bees after 6-day feeding treatments. New emerged bees in groups of 50 bees fed with 50% sucrose syrup and bee pollen (S + P), 50% sucrose syrup and bee pollen with addition of SYM broth (S + SYM-P) and 50% sucrose syrup and CK-CsC fermented bee pollen broth (S + CK-CsC-P). Each point represents the cumulative survival rate (N = 4 cages from four colonies). There is no significant difference between survival curves (Log-Rank test: *p* = 0.524). (**b**) Gene expression profile of honey bees after 6-day feeding treatments. C: control, bees feeding with 50% sucrose syrup and bee pollen with addition of SYM broth; F: bees feeding with 50% sucrose syrup and CK-CsC fermented bee pollen broth. The relative gene expression was analyzed using the 2^−ΔΔ*C*T^ method. Data represent the mean of four replicates, with error bars indicating the standard deviation. Bar with the label * is significantly different (*p* < 0.05) accordingly to the Mann-Whitney U test.

Newly emerged adult bees were divided into three groups according to how they were fed CK–CsC fermented in a SYM culture medium: 50% sucrose syrup and bee pollen (S + P), 50% sucrose syrup and bee pollen with added SYM broth (S + SYM-P), and 50% sucrose syrup and CK-CsC fermented bee pollen broth (S + CK–CsC-P). Six days later, the survival rates in the S + P, S + SYM-P, and S + CK–CsC-P groups were 98%, 99%, and 98%, respectively (Figure 3a, χ^2^_2, 600_ = 1.3, *p* > 0.05). This indicates that pollen fermented by CK–CsC is not toxic to honey bees.

With regard to the addition of CK–CsC to pollen, the originally solid pollen became a viscous liquid after 4 days of fermentation. This indicates that the CK–CsC reacted with the pollen. Compared to the honey bees fed 50% sugar syrup and pollen with added SYM broth (the S + SYM-P group), those that fed for 6 days on the pollen broth that was fermented for 4 days (the S + CK–CsC-P group) expressed 15 and 5 times more *mrjp1* and *vg* (Z = −1.299, *p* > 0.05 for *mrjp1*; Z= −2.3067, *p* < 0.05 for *vg*; Figure 3b). The stimulation effect is attributable to the secretion of extracellular enzymes such as cellulase and pectinase by the CK–CsC (Supplementary Figure S1). Specifically, these enzymes degraded the pollen cell walls, allowing the honey bees to absorb the nutrients more easily and promote their nutrient gene expression. Simultaneously, the consumption of the pollen fermentation broth increased the expression of apidaecin and hymenoptaecin by 1.8 times, respectively (Z = −0.1443, *p* > 0.05 for apidaecin; Z = 0.433, *p* > 0.05 for hymenoptaecin; Figure 3b). This immune gene expression increase is likely ascribable to the pollen contents or pollen-borne microbes [67,69,70].

### 3.5. Gut Microflora Changes

Honey bees digest and metabolize carbohydrates with the enzymes they secrete as well as their gut microbes [66]. This premise is similar to that presented by Zheng, et al. [24], who confirmed that *Gilliamella apicola* isolated from honey bee guts can utilize arabinose, mannose, rhamnose, and xylose, sugars that honey bees cannot metabolize. The bacterium is speculated to promote honey bees’ dietary sugar tolerance. The EPS extract obtained from fermentation with CK–CsC led to a high mortality rate that would have been lower if the honey bees had fed on bee bread at the same time. Therefore, the microflora in the pollen were speculated to assist with the metabolism of the crude EPS extract. Further analysis was conducted to determine whether the gut microflora of the honey bees fed syrup containing crude EPS extract (E group) differed from that of the honey bees fed both syrup containing crude EPS extract and bee pollen cakes (E + P group).

**Figure 4:**
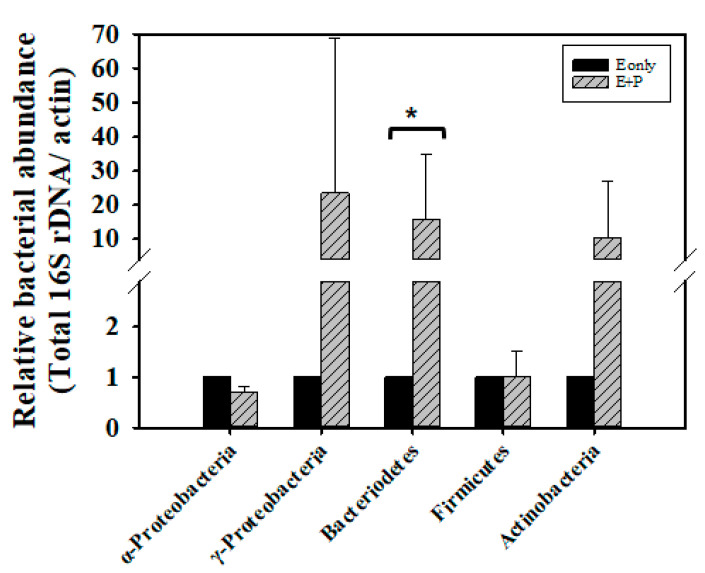
Bacterial abundance in the guts of honey bees fed with EPS and bee pollen. The bacterial loads of α-Proteobacillus, γ-Proteobacillus, Bacteriodetes, Firmicutes and Actinobacteria in the guts of bees were assessed through quantitative polymerase chain reaction (qPCR). DNA samples obtained from the guts of 7-day-old workers fed with 50% sucrose syrup containing 1% EPS (E only) or 50% sucrose syrup containing 1% EPS and bee pollen (E + P), were prepared for qPCR. The relative bacterial abundance was analyzed using the 2^−^^ΔΔCT^ method. Data represent the mean of four repeats (10 guts for each repeat), with error bars indicating the standard deviation. Bar with the label * is significantly different (*p* < 0.05) accordingly to the Mann-Whitney U test.

Newly emerged adult bees obtain food from honeycombs, such as honey and bee bread; this contributes to gut microflora establishment [71]. In our experiment, we collected 12-h-old newly emerged bees which have obtained microflora from food stored in honeycomb, and fed these newly emerged bees syrup containing crude EPS extract for 3 days, after which we determined the counts of midgut and hindgut bacteria. No differences in total bacterial count were found between the E group and E + P group (Z = 0, *p* > 0.05; Supplementary Figure S4). However, the E + P group exhibited 23.5 times the amount of γ-Proteobacteria (Z = −1.0445, *p* > 0.05), 15.9 times the amount of Bacteroidetes (Z = 2.0889, *p* < 0.05), and 10.3 times the amount of Actinobacteria (Z = 0.6267, *p* > 0.05) bacteria of the E group. Nevertheless, only the amount of Bacteroidetes showed statistically significant difference. The amount of γ-Proteobacteria and Actinobacteria show wide variation from the four tested colonies, causing non-significant difference statistically. The counts of α-Proteobacteria (Z= −1.0445, *p* > 0.05) and Firmicutes (Z = −0.7178, *p* > 0.05) did not show difference between the E group and E + P group (Figure 4).

Studies on genome sequences have revealed the secretion of glycoside hydrolases by bacteria from the γ-Proteobacteria, Bacteroidetes, Actinobacteria, and Firmicutes, which have also been speculated to have the ability to metabolize polysaccharides [24,25,72]. This premise is consistent with our findings. Although we did not observe an increase in the Firmicutes count in the E + P group, we noted that the counts of *Lactobacillus kunkeei* increased by 136.4 times (Z = 1.2534, *p* > 0.05), through the intake of pollen (Supplementary Figure S4). Despite the finding that the difference of *L. kunkeei* between the E group and E + P group is not statistically significant, bees from four tested colonies show the trend of increased amount of *L. kunkeei* in the E + P group. This phenomenon also matches with previous studies which show *L. kunkeei* is a dominant bacterium in bee pollen [14,73]. The present results further highlight the vital role of honey bee gut microbes in carbohydrate metabolism.

## 4. Conclusions

This study was an exploration of the effects of broth fermented with *A. melanogenum* CK–CsC isolated from bee bread, on survival, expression of nutrient and immune related genes, and gut microbiota of honey bees. The crude EPS extract was toxic to the honey bees, causing a high mortality rate that would have been reduced if the honey bees had simultaneously fed on bee bread pollen. Comparison of the midgut and hindgut microflora of the honey bees fed crude EPS extract and the honey bees fed crude EPS extract with pollen revealed that feeding on pollen increased the populations of γ-Proteobacteria, Bacteroidetes, and Actinobacteria in the gut. Bacteria in these phyla may help honey bees break down crude EPS extract. Furthermore, in honey bees fed the CK–CsC fermentation broth in SYM medium or the pollen fermentation broth, nutrient gene expression was stimulated. However, the expression of antibacterial peptide genes apidaecin and hymenoptaecin was not stimulated by the consumption of broth fermented with the CK-CsC strain. Their expression was only promoted when the honey bees fed on pollen fermented with CK–CsC. This highlights the importance of bee pollen to honey bee physiology and the maintenance of honey bee gut microflora, along with the major contribution of gut bacteria to host metabolism. These findings serve as a reference on *A. melanogenum* fermentation for scientists studying honey bee health.

## Figures and Tables

**Table 1:** Characterization of the *A. malanogenum* CK–CsC fermented broth.

Time (Day)	pH	Protein (mg/mL)	Biomass (mg/mL)	Exopolysaccharides (mg/mL)
1	5.8 ± 0.6	5.5 ± 0.0	21.4 ± 3.6	4.4 ± 1.6
2	5.4 ± 0.6	12.4 ± 0.4	26.4 ± 2.3	6.7 ± 2.7
3	4.0 ± 0.2	18.2 ± 0.7	25.0 ± 3.8	10.6 ± 2.1
4	3.5 ± 0.1	20.0 ± 0.3	33.9 ± 3.3	14.1 ± 1.9
5	3.1± 0.0	21.6 ± 0.6	43.4 ± 4.5	15.8 ± 2.1

## Data Availability

The data that support the findings of this study are available from the corresponding author on request.

## Supplementary Materials

The following are available online at https://www.mdpi.com/article/10.3390/jof7070508/s1, Figure S1: Pectinase (A) and cellulase (B) activity of the CK-CsC. Figure S2: The cumulative survival of honey bees after 6-day feeding treatments. Figure S3: Pollen fermented by *Aureobasidium melanogenum* CK–CsC for four days. Figure S4: Bacterial abundance in the guts of honey bees fed with EPS and bee pollen. Table S1: Primers used for fungi classification and bacterial quantification. Table S2: Primers used for quantification of honey bee gene expression. Table S3: The information of the fungi isolated in this study.
