# Supplementary material for: A Potential Fungal Probiotic Aureobasidium melanogenum CK-CsC for the Western Honey Bee, Apis mellifera
Source: J Fungi (Basel). 2021 Jun 25;7(7):508. doi: 10.3390/jof7070508 (PMC8306588; doi:10.3390/jof7070508)
Supplement: Supplementary file 1 [file jof-07-00508-s001.zip › jof-1270766-supplementary.pdf]

Supplementary Table S1. Primers used for fungi classification and bacterial quantification

Target	Primer	Primer sequence (5' to 3')	Tm (°C)	Source
For fungi classification				
Internal transcribed spacer (ITS)	ITS 1	TCCGTAGGTGAACCTGC	50	Raja et al. 2017.
	ITS 4	TCCTCCGCTTATTGATATGC		
For bacterial quantification				
Total bacteria	16S-27F	AGAGTTTGATCCTGGCTCAG	59	Raymann et al. 2017.
	16S-355R	GCTGCCTCCCGTAGGAGT		
Lactobacillus kunkeei	LKunkeei F	TGGGTAACCTGCCCCGAAG	59	In this study.
	LKunkeei R	TCTTGGTGGGCTTTTATCTCAC		
α –Proteobacteria	F	CIAGTGTAGAGGTGAAATT	55	Bacchetti De Gregoris, et al. 2011.
	R	CCCCGTCAATTCCTTTGAGTT		
γ – Proteobacteria	F	TCGTCAGCTCGTGTYGTGA	55	
	R	CGTAAGGGCCATGATG		
Bacteroidetes	F	CRAACAGGATTAGATACCCT	55	
	R	GGTAAGGTTCCTCGCGTAT		
Firmicutes	F	TGAAACTYAAAGGAATTGACG	55	
	R	ACCATGCACCACCTGTC		
Actinobacteria	F	TACGGCCGCAAGGCTA	55	
	R	TCRTCCCCACCTTCCTCC		

Supplementary Table S2. Primers used for quantification of honey bee gene expression

Target	Primer	Primer sequence (5' to 3')	Tm (ºC)	Source
**mrjp1**	F	CACAGCCCAAGATGGAATTT	59	Wu et al., 2017.
	R	AAGAGGACGCCACTCTTTGA		
**vg**	F	GTTGGAGAGCAACATGCAGA	59	Zheng et al. 2017.
	R	TCGATCCATTCCTTGATGGT		
**apidaecin**	F	TAGTCGCGGTATTTGGGAAT	59	In this study.
	R	TTAGATTCGCGGATGAGGT		
**hymenoptaecin**	F	CTCTTCTGTGCCGTTGCATA	59	
	R	GCGTCTCCTGTCATTCCATT		
**actin**	F	TGCCAACACTGTCCTTTCTG	55	Scharlaken et al. 2008.
	R	AGAATTGACCCACCAATCCA		
**rpS18**	F	GATTCCCGATTGGTTTTTGA	55	
	R	CCCAATAATGACGCAAACCT		

Supplementary Table S3. The information of the fungi isolated in this study

Strain	Accession number	The Matched Species	Identity (%)
**CK–CsAf**	**MT890015**	***Aspergillus flavus***	**100**
**CK–CsC**	**MT791349**	***Aureobasidium melanogenum***	**100**
**CK–CsCt**	**MT890016**	***Cladosporium tenuissimum***	**99.8**
**CK–CsP**	**MT890014**	***Penicillium oxalicum***	**98.7**

(A)

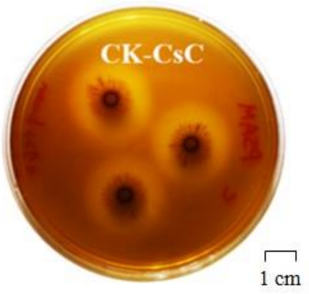

(B)

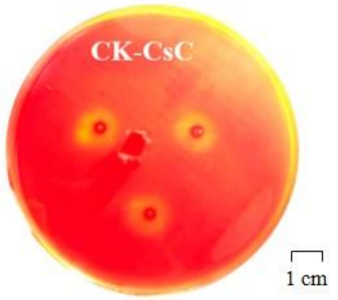

Supplementary Figure S1. Pectinase (A) and cellulase (B) activity of the CK-CsC. The CK-CsC were inoculated on pectinase screening agar and cellulase screening agar, and agar plates were incubated at 28°C for one week. Then Lugol solution and congo red dye were used for staining, respectively. A clear halo zone around the colonies indicates the ability of the CK-CsC to produce pectinase and cellulase.

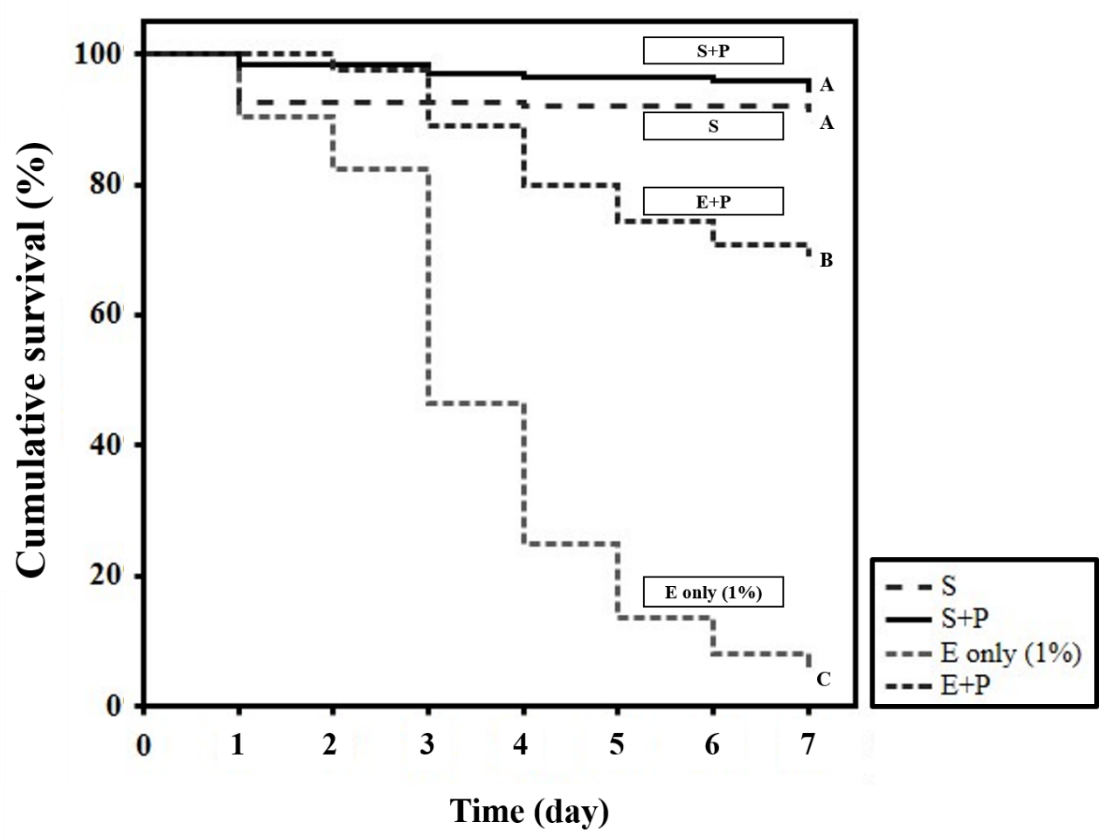

Supplementary Figure S2. The cumulative survival of honey bees after 6-day feeding treatments. New emerged bees in the groups of 50 bees fed with 50% sucrose syrup (S), 50% sucrose syrup and bee pollen (S+P), 1% exopolysaccharide crude extract in 50% sucrose syrup (E only, 1%) and 1% exopolysaccharide crude extract in 50% sucrose syrup and bee pollen (E+P). Worker bees were incubated at 34°C. Each point represents the cumulative survival rate (N = 4 cages from four colonies). Different letters are significantly different ($P < 0.05$) accordingly to the Log-Rank tests.

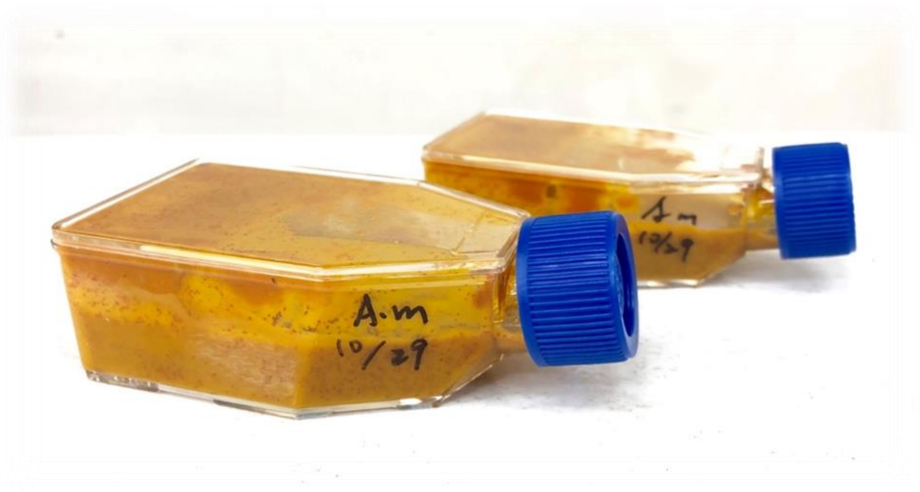

Supplementary Figure S3. Pollen fermented by *Aureobasidium melanogenum* CK–CsC for four days. 10^7 spores of the CK-CsC were inoculated into 10 mL of YPD medium (seed culture) with shaking at 28°C for one day. Then the 10 mL of seed culture was added to 30 g of pollen, mixed well, then placed at 28°C for 4 day-solid fermentation.

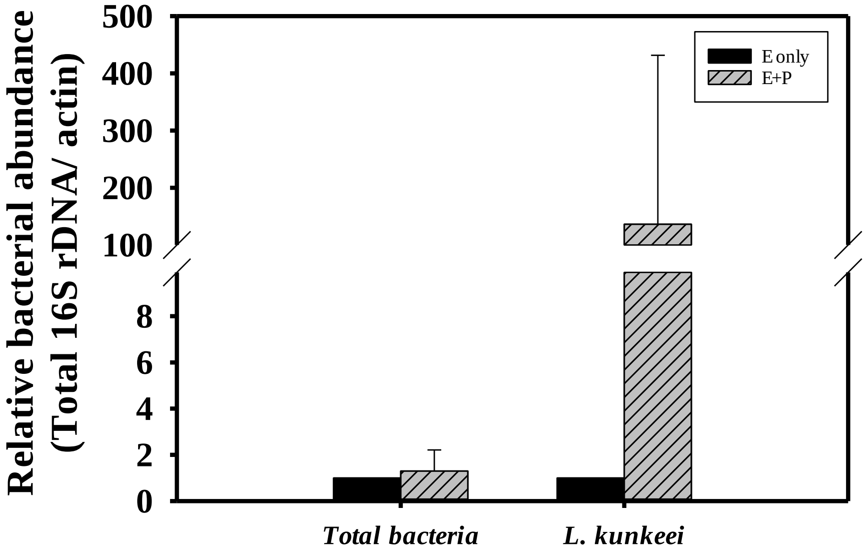

Supplementary Figure S4. Bacterial abundance in the guts of honey bees fed with EPS and bee pollen. The bacterial loads of total bacteria and *Lactobacillus kunkeei* in the guts of bees were assessed through quantitative polymerase chain reaction (qPCR). DNA samples obtained from the guts of 7-day-old workers fed with 50% sucrose syrup containing 1% EPS (E only) or 50% sucrose syrup containing 1% EPS and bee pollen (E+P), were prepared for qPCR. The relative bacterial abundance was analyzed using the $2^{-\Delta\Delta CT}$ method. Data represent the mean of four repeats (10 guts for each repeat), with error bars indicating the standard deviation.
